# Early Detection of Dysphagia Signs in Parkinson’s Disease: An Artificial Intelligence-Based Approach Using Non-Invasive Sensors

**DOI:** 10.3390/s25226834

**Published:** 2025-11-08

**Authors:** Michele Antonio Gazzanti Pugliese di Cotrone, Nidà Farooq Akhtar, Martina Patera, Silvia Gallo, Umberto Mosca, Marco Ghislieri, Claudia Ferraris, Antonio Suppa, Carlo Alberto Artusi, Alessandro Zampogna, Gianluca Amprimo, Gabriele Imbalzano, Serena Cerfoglio, Veronica Cimolin, Luigi Borzì, Gabriella Olmo, Fernanda Irrera

**Affiliations:** 1 Department of Information Engineering, Electronics and Telecommunications, Sapienza University of Rome, 00185 Rome, Italy; nida.farooq@estudiantat.upc.edu (N.F.A.); fernanda.irrera@uniroma1.it (F.I.); 2 Department of Human Neurosciences, Sapienza University of Rome, 00185 Rome, Italy; martina.patera@uniroma1.it (M.P.); antonio.suppa@uniroma1.it (A.S.); alessandro.zampogna@uniroma1.it (A.Z.); 3 Department of Electronic Engineering, Universitat Politècnica de Catalunya, 08034 Barcelona, Spain; 4 Department of Neurosciences “Rita Levi Montalcini”, University of Turin, 10124 Turin, Italy; sil.gallo@unito.it (S.G.); carloalberto.artusi@unito.it (C.A.A.); gabriele.imbalzano@unito.it (G.I.); 5 Department of Control and Computer Engineering, Politecnico di Torino, 10129 Turin, Italy; umberto.mosca@polito.it (U.M.); gianluca.amprimo@polito.it (G.A.); luigi.borzi@polito.it (L.B.); gabriella.olmo@polito.it (G.O.); 6 PolitoBIOMed Lab, Politecnico di Torino, 10129 Turin, Italy; marco.ghislieri@polito.it; 7 Department of Electronics and Telecommunications, Politecnico di Torino, 10129 Turin, Italy; 8 Institute of Electronics, Computer and Telecommunication Engineering (IEIIT), Consiglio Nazionale delle Ricerche (CNR), 10135 Turin, Italy; claudia.ferraris@cnr.it; 9 IRCCS Neuromed, 86077 Pozzilli, Italy; 10 Department of Electronics, Information and Bioengineering, Politecnico di Milano, 20133 Milan, Italy; serena.cerfoglio@polimi.it (S.C.); veronica.cimolin@polimi.it (V.C.); 11 IRCCS Istituto Auxologico Italiano, San Giuseppe Hospital, Strada Luigi Cadorna 90, 28824 Oggebbio, Italy

**Keywords:** wearable sensors, artificial intelligence, surface electromyography, swallowing features, principal component analysis, dysphagia signs, Parkinson’s Disease

## Abstract

The present study evaluates the effectiveness of a non-invasive wearable sensor system, combining accelerometers, surface electromyography, and artificial intelligence, to objectively characterize swallowing in elderly individuals affected by Parkinson’s Disease, without clinically manifested dysphagia. A cohort of patients and healthy control subjects performed the same swallowing test protocol, including tasks with different viscosity boluses, positioning a commercial adhesive grid of High-Density surface Electromyography (HD-sEMG) electrodes on the submental muscle and a triaxial accelerometer over the thyroid cartilage. Relevant temporal and spectral features were extracted from electromyography data. Proper filtering and processing by machine learning and Principal Component Analysis allowed identification of two distinct clusters of subjects, one predominantly composed of controls with just a few patients, the other mostly crowded by patients. Excellent classification performances were achieved (accuracy = 83.3%, precision = 79.0%, recall = 90.7%, F1-score = 84.5%, Cohen’s kappa = 0.67), revealing consistent differences in muscle activation patterns among subjects, even in the absence of clinically diagnosed dysphagia. These results support the feasibility of wearable sensor-based assessment as a reliable and non-invasive tool for the early detection of subclinical swallowing dysfunction in Parkinson’s Disease.

## 1. Introduction

Swallowing is recognized as a complex, yet stereotyped motor sequence that requires the finely tuned bilateral coordination of several muscles distributed across the oral cavity, pharynx, larynx, and esophagus [1]. Traditionally, the swallowing process has been described in three distinct phases: an initial oral phase, under voluntary control, followed by the pharyngeal and esophageal phases, which are involuntary and regulated by highly integrated neural reflex mechanisms across the motor cortex, the basal ganglia, and the brainstem [2]. Dysphagia, or difficulty in swallowing, is a common symptom in elderly individuals (and, more specifically, in patients with a neurodegenerative disorder), significantly affecting quality of life and increasing the risk of aspiration pneumonia, malnutrition, and dehydration [3]. Swallowing impairment may be due to several conditions, often compromising the complex interactions between central and peripheral mechanisms controlling swallowing. In Parkinson’s Disease (PD), neurodegeneration affects both cortical-basal ganglia circuits and brainstem swallowing centers, leading to subtle alterations in swallowing coordination even before overt clinical dysphagia is diagnosed [4,5,6]. Studies report that up to 80% of PD patients may develop some form of dysphagia throughout the course of the disease [6,7], although patient awareness of swallowing difficulties is generally very limited. A meta-analysis reported a pooled prevalence of oropharyngeal dysphagia among PD patients of 35% when based on subjective and clinical evaluations, increasing to 82% after objective assessment [8]. Aspiration pneumonia is a major cause of death in PD [9]; hence, the early identification of features related to dysphagia could help implementing timely therapeutic interventions, significantly reducing morbidity and mortality among patients. Conventional techniques for the diagnosis of dysphagia are based on videofluoroscopic swallow study (VFSS) and fiberoptic endoscopy evaluation of swallowing (FEES) [10,11], which are resource-intensive and carry procedural risks, being at the same time invasive and burdensome for patients, thus limiting their routine use and contributing to the underdiagnosis and undertreatment of dysphagia. Conversely, for widespread screening and follow-up, alternative non-invasive and non-ionizing screening framework monitoring approaches are desired. For this reason, in recent decades, non-invasive methods using surface electromyography (sEMG) and/or inertial measurement units (IMUs) have been investigated as complementary (and, in a few cases, alternative) ways to monitor swallowing function [12,13,14,15,16], with a few papers published in the last year [17,18,19], reflecting the growing interest in the field. The review paper by Wu et al. (2024) [17] points out the strong need for standardized, non-invasive tools to replace current gold-standard methods like VFSS and FEES and highlights that surface sensors such as sEMG and accelerometers are promising for real-time and long-term monitoring of swallowing. In the research paper by Suarez-Patiño et al. (2024) [18], the authors integrate synchronized sEMG and VFSS to assess oropharyngeal dysphagia, which involves the anatomical tracking of bolus transit through structures such as the mandibular line, vallecula, and upper esophageal sphincter using VFSS, while mapping muscle activation timing via sEMG. Roldán-Vasco et al. (2023) [15] analyzed multichannel sEMG, extracting time, frequency, and non-linear features to classify swallowing disorders. Hong et al. (2025) [19] developed a custom soft, high-density (HD) 64-channel sEMG (HD-sEMG) array through a meticulous sensor integration and electrode fabrication. They employed a machine learning pipeline for accurate and non-invasive dysphagia assessment in elderly healthy subjects and stroke patients, reporting excellent classification accuracy with advanced preprocessing, feature extraction, and dimensionality reduction via Linear Discriminant Analysis (LDA), followed by Random Forest classification. It is worth noticing that, to this date, the attention of most researchers has been focused on patients who already had the diagnosis of dysphagia [15,17,18], while a more challenging target would be the identification of features related to the disease, years before its clinical assessment.

In our study, we developed a non-invasive and well-tolerated method based on processing sEMG data recorded by a HD commercial adhesive matrix of 32 electrodes positioned on the submental muscle and using offline analysis of the biopotentials in time and frequency domains. A triaxial accelerometer was positioned over the thyroid cartilage (Adam’s apple) and a video recording was also used for the system’s initial setting. sEMG signals captured the muscle activity involved in swallowing, from which time and frequency domain features were extracted as candidate biomarkers of swallowing dysfunction. The accelerometer sensor could track laryngeal linear acceleration during swallowing, offering complementary kinematic information. The video recording served to overview the actions during the tests.

A cohort of fifteen PD patients receiving dopaminergic treatment was enrolled, all without a clinically overt dysphagia. Patients underwent clinical assessments evaluating overall disease severity and functional impairment. Seven healthy, age-matched controls participated in the study. All the enrolled subjects performed the same sequence of swallowing tests (with liquid water, gelled water, bread), under the supervision of neurologists. Milestones of this work are to look for direct correlations between sEMG-derived swallowing features and the PD scores (or other aspecific scores), and to identify a set of sEMG features related to the swallowing characteristics dividing patients into two distinct clusters: one cluster closely aligned with healthy subjects and another diverging from controls.

Contrary to previous studies, our system relies on sEMG signals acquired through commercially available electrodes, complemented by a triaxial accelerometer and video recordings. It specifically targets PD patients without a diagnosis of dysphagia, filling an important gap in early detection of subclinical swallowing dysfunction. It offers a complete and practical approach that includes signal segmentation, feature extraction, and simple machine learning models that work across different swallowing tasks. We emphasize temporal and spectral features and integrate cluster analysis to capture subclinical swallowing changes, expanding the diagnostic scope beyond classification and proposing a more accessible, non-radiative screening framework.

## 2. Materials and Methods

### 2.1. Set-Up

**Figure 1:**
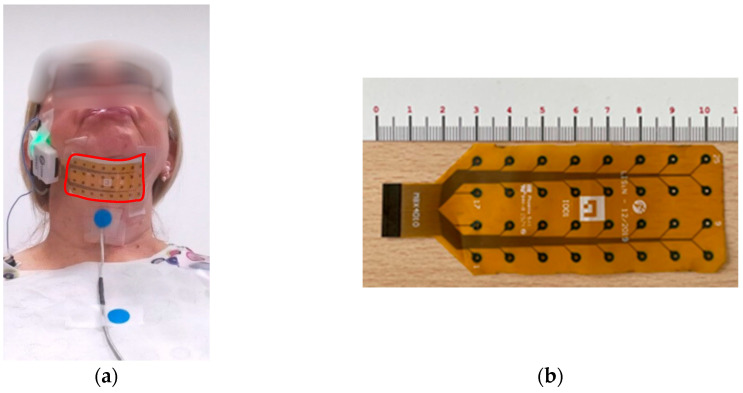
(**a**) a picture of the overall system, with the HD-sEMG sensor matrix (highlighted with a red contour) positioned over the suprahyoid muscles using a clavicular reference electrode and a miniaturized accelerometer positioned on the thyroid cartilage; (**b**) the 32-channel HD-sEMG sensor matrix.

The study adopts a multimodal approach integrating wearable sensors and video analysis. The sensor set-up includes a commercial [20] 32-channel monopolar HD-sEMG grid (4 rows × 8 columns, inter-electrode distance: 10 mm) placed over the suprahyoid muscles. To minimize potential issues with sensor adhesion, especially in the presence of facial hair, patients were asked to shave the submental area before the recording session. sEMG signals were acquired in a monopolar configuration at 2048 Hz with 16-bit resolution (MEACS, ReC Bioengineering and LISiN, Politecnico di Torino, Turin, Italy) [21] using a clavicular reference electrode. A miniaturized triaxial accelerometer (LIS344ALH, STMicroelectronics, The Netherlands, Amsterdam) was positioned on the thyroid cartilage using adhesive tape. The accelerometer sensor sampled at 1024 Hz. It was integrated to minimize discomfort and optimize patient compliance, being placed on the laryngeal prominence (Adam’s apple) [22]. The synchronization system consists of external SyncU modules that communicate over a dedicated, low-latency radio link as detailed by Cerone et al. [23]. One module operates as the transmitter (SyncU Tx, ReC Bioengineering and LISiN, Politecnico di Torino, Turin, Italy), while the others two act as receivers (SyncU Rx, ReC Bioengineering and LISiN, Politecnico di Torino, Turin, Italy) connected to the HD-sEMG acquisition unit and the accelerometer requiring synchronization. A picture of the overall system is reported in Figure 1a, where in Figure 1b the HD-sEMG sensor matrix is depicted. A Kinect Azure camera simultaneously captured video data using two colored adhesive markers—one on the thyroid cartilage and another on the sternum. A common synchronization pulse was used to realign offline videos, accelerometric data, and sEMG signals [23].

### 2.2. Participants

A total of 15 PD patients (4 females and 11 males; age 69.5 ± 7.5 years—mean ± standard deviation) and 7 healthy controls (HC) (4 females and 3 males; age 67 ± 4.8 years) were recruited at the Movement Disorders outpatient clinic of Sapienza University of Rome and at the Department of Neuroscience “Rita Levi Montalcini” of the University of Turin, Italy. Patients were enrolled according to the following inclusion criteria: diagnosis of PD based on standardized Movement Disorder Society (MDS) criteria [24] and moderate-to-advanced disease stage corresponding to a Hoehn and Yahr score between 2.5 and 4 [25]. Exclusion criteria were: diagnosis of atypical, vascular, or drug-induced parkinsonism; presence of severe dementia, defined as a Montreal Cognitive Assessment (MoCA) score ≤ 21 [26]; inability to provide informed consent; and severe non-neurological structural or functional disorders affecting swallowing or phonation.

All patients underwent a clinical assessment using a battery of standardized scales. Moreover, patients underwent the Hoehn and Yahr scale, the Movement Disorder Society-Unified Parkinson’s Disease Rating Scale (MDS-UPDRS) [27], the MoCA, and the Frontal Assessment Battery (FAB) [28]. The subjective perception of swallowing function was investigated through the Swallowing Disturbance Questionnaire (SDQ) [29].

Table 1 summarizes the main demographic and clinical information. The patient IDs are indicated as PTx for those patients coming from Turin and as PRx for those coming from Rome. All the enrolled subjects have given written informed consent to the study which was approved by the institutional review board (“Comitato Etico Territoriale (CET) interaziendale AOU città della salute e della scienza di Torino” Protocol 0080563 No. 512/2023; approved on 17 June 2024), following the Declaration of Helsinki.

### 2.3. Experimental Protocol

Patients were assessed in two sessions: the first consisted of the clinical evaluation conducted by two movement disorders experts, and the second involved the recording sessions for swallowing tasks. To minimize the influence of the dopaminergic state, all swallowing recordings in PD patients were performed during the ON phase of dopaminergic therapy (i.e., approximately one hour after medication intake). For each patient, the daily levodopa equivalent dose (LEDD) was calculated according to standardized procedures. During the swallowing tasks, patients sat upright with a stable head position. They performed a sequence of swallowing tests, using bolus with increasing viscosity, with a similar approach to the works of Hong et al. [17] and Roldán-Vasco et al. [15]. The swallowing sequence consisted of the following tasks, in the following order: (a) the Water Task (WT) with 5 mL water; (b) the Gelled water Task (GT) with 5 mL gelled water; and (c) the Solid bolus Task (ST) using 3.5 g of bread. Each task was repeated three times (three trials). The oral intake (for the WT and the GT) or the mastication phase (for the ST) was followed by a rest phase, during which patients held the bolus in the oral cavity until instructed to swallow. Each swallowing trial lasted approximately 10–20 s, while the entire sequence of tasks required about 15 min on average, including patient seating and instruction, as well as rest periods between trials and between tasks to minimize fatigue and performance decline.

To minimize placement bias for both HD-sEMG and accelerometers, a single experienced operator positioned all sensors for every participant under a standardized positioning protocol. Susceptibility to motion artifacts was further limited because the task was performed with participants seated at a table.

Seven adult healthy controls performed the same tests in the same conditions; their demographic data and SDQ scores are reported in Table 2. The experimental procedures and set-up were carried out in collaboration with a team of engineers from Sapienza University of Rome, Politecnico di Torino, Politecnico di Milano, University of Turin, and National Research Council (CNR).

### 2.4. Identification of the Swallowing Acts

**Figure 2:**
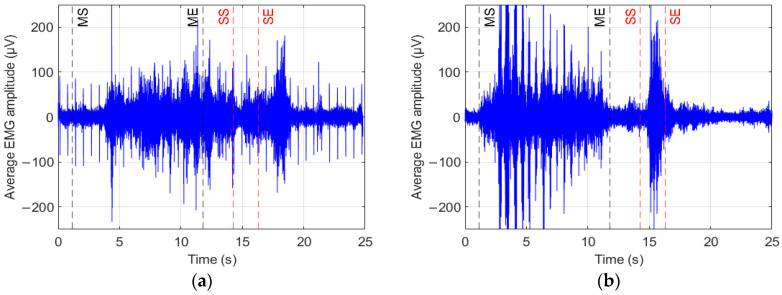
Typical traces of the sEMG averaged over the 32 channels recorded during a trial of the ST, which includes the different phases after bolus intake (mastication start (MS), mastication end (ME), swallowing start (SS), swallowing end (SE)). Traces refer to a healthy control (**a**) and a PD patient (**b**) of the sample population.

To isolate the swallow intervals in all the tasks (water, gel, bread), portions of signals related to oral intake, permanence of the bolus in the mouth, and mastication were removed, as they were not of interest for this study. This was achieved by careful video analysis. sEMG signals were first notch-filtered at 50 Hz to remove power line interference and then band-pass filtered between 1 and 400 Hz (4th-order Butterworth, zero-phase). Accelerometer signals from each axis were band-pass filtered between 0.1 and 5 Hz (4th-order Butterworth, zero-phase). Figure 2 shows a typical trace of the sEMG averaged over the 32 channels recorded during a trial of the ST, which includes the phases of: mastication (start and end, respectively, MS and ME), rest, swallowing (start and end, respectively, SS and SE), rest. Mastication phases were manually identified, while swallowing onset and offset were detected using the automated method described later in this section. In that figure, the trace referring to a healthy control (a) and a patient (b) are displayed. Various normalization approaches were applied to minimize inter-subject and inter-trial variability of the sEMG signal. Specifically, we implemented z-score normalization, which standardizes signals by dividing them by their standard deviation, resulting in unit variance. Robust normalization was also used, involving the subtraction of the median value and scaling by the interquartile range (IQR), thus reducing sensitivity to outliers.

After normalization, we segmented both sEMG and accelerometer signals in overlapped windows with 0.5 s length and 0.3 s overlap, empirically selected. A number of energy-related features were extracted from both the sEMG and accelerometer signals in each window and then fused together. We detected the peak of the fused feature vector across sEMG and accelerometer signals for each trial. For subsequent analysis, we defined 2 s epochs centered on each peak (tstart = tpeak − 1 s; tend = tpeak + 1 s), ensuring all segments had equal length.

### 2.5. Feature Extraction and Correlations with PD Scores

Signals from the HD-sEMG matrix, accelerometer, and video recordings were synchronized and processed accordingly. Accelerometer and sEMG data were preprocessed using the same filtering procedures described in the previous subsection. sEMG signals were averaged over the 32 channels.

For WT, GT, and ST swallowing segments, we extracted the following time-domain features derived from the sEMG signals, which have shown diagnostic value in dysphagia screening in the literature [15]: the root mean square (*RMS_val_*), defined as the square root of the arithmetic mean of the squared sEMG signal (Equation (1)), and the waveform length (*WL_val_*), which is the cumulative length of the signal waveform over time, defined as the sum of the absolute differences between sEMG signal samples across time (Equation (2)). An RMS-based asymmetry index (*AS_val_*) between the right and left sides of the HD-sEMG matrix (respectively, the top half and the bottom half in Figure 1a) was also calculated as the difference between the mean RMS of the left and right sides of the 4 × 8 HD-sEMG matrix across time (Equation (3)). The use of this asymmetry feature is supported by recent work on the Degree of Symmetry (DOS) indicator proposed by Hong et al. [19], which also relies on side-specific RMS measurements to quantify bilateral coordination during swallowing. In the following equations, *sEMG_i_* denotes the sEMG signal at sample *i* and *N* is the number of samples. *RMSleft* and *RMSright* are the RMS of the average values of the left side and right side in the HD-sEMG matrix computed within the selected 2 s epoch.

(1) $${RMS}_{val}=\sqrt{\frac{1}{N}\sum_{i=1}^{N}{sEMG}_{i}^{2}}$$

(2) $${WL}_{val}=\sum_{i=1}^{N-1}\left|{sEMG}_{i+1}-s{EMG}_{i}\right|$$

(3) $${AS}_{val}={sRMS}_{left}-{sRMS}_{right}$$

Additionally, frequency-domain analysis was performed on the same averaged *sEMG* segments after applying the Fast Fourier Transform (FFT). The FFT length was set equal to the segment length, resulting in a frequency resolution determined by the sampling frequency and the number of samples per segment. The following sEMG-derived features, commonly reported in the literature [15], were then calculated: peak frequency (*PF_val_*), defined as the frequency corresponding to the maximum amplitude of the FFT (Equation (4)); the average frequency (*AF_val_*), defined as the weighted average of the FFT frequencies (Equation (5)); and the total power (*TP_val_*), computed as the sum of the squared FFT magnitudes (Equation (6)), representing the total spectral energy of the segment. Since all segments had identical length, no additional normalization was applied, allowing *TP* values to be directly comparable across trials and subjects.

(4) $${PF}_{val}=max({sEMG}_{FFT})$$

(5) $${AF}_{val}=\frac{\Sigma_{i}f_{i}{s{EMG}_{FFT}}_{i}}{\Sigma_{i}{s{EMG}_{FFT}}_{i}}$$

(6) $${TP}_{val}=\Sigma_{i}({sEMG}_{FFT_{i}}{)}^{2}$$

In these expressions, “*sEMG_FFT_*” denotes the magnitude of the Fourier transform of the sEMG signal at each discrete frequency bin, and fi are the corresponding frequency bins, which represent the signal’s energy contribution at those frequencies.

(7) x_norm=(x−x_min)/(x_max−x_min)

As for the normalization procedure, it was performed as follows: min-max normalization (Equation (7)) was applied to all the features except *AS_val_*, while z-score normalization (Equation (8)) was applied to the *AS_val_* feature to preserve its directionality. *x* is the original value, *x_min* and *x_max* are the minimum and maximum values of the dataset, and *x_norm* is the normalized value between 0 and 1.

(8) z_norm=(x−μ)/σ

*x* is the original value, *μ* is the mean of the dataset, *σ* is the standard deviation of the dataset, and *z_norm* is the normalized value.

(9) $$r=\frac{\sum_{i=1}^{n}\left(X_{i}-\bar{X})(Y_{i}-\bar{Y}\right)}{\sqrt{\sum_{i=1}^{n}(X_{i}-\bar{X}{)}^{2}}\;\;\sqrt{\sum_{i=1}^{n}(Y_{i}-\bar{Y}{)}^{2}}}$$

Lastly the correlation between variables was performed using the Pearson correlation coefficient *r*. Where *X_i_* and *Y_i_* are individual sample values, $\bar{x}$ and *Ῡ* are the sample means of *X* and *Y*, and n is the number of paired observations. The Pearson correlation coefficient *r* quantifies the linear relationship between two variables, ranging from −1 (perfect negative) to +1 (perfect positive), with 0 indicating no linear correlation. The *p*-value tests the null hypothesis that there is no linear correlation. In this study, a *p*-value < 0.05 was considered statistically significant.

### 2.6. Principal Component Analysis

To provide a more comprehensive characterization of the swallowing activity, a broader set of features was computed. First, we ensured that the input segments had the same length across all subjects and tasks, fixing a duration of two seconds centered on the identified swallowing peak. In this analysis, sEMG signals underwent the same preprocessing and segmentation procedures described in Section 2.4. The signals from each of the 32 channels were then normalized using min-max scaling, which reduces inter-subject variability due to skin conductivity, sweating, or electrode placement. Time-domain features were extracted from the normalized signals and included measures such as mean absolute value, waveform length, zero crossings, slope sign changes, integrated sEMG, statistical descriptors (mean, variance, skewness, kurtosis), and dynamic properties like amplitude change and complexity indices. Frequency-domain features were computed on the non-normalized signals and included mean and median frequency, spectral peak, total power, and various entropy-based features [15,30].

Spectral energy was also computed using the short-time Fourier transform, which analyzes how the frequency content of the signal evolves over time by applying the Fourier transform on overlapping windows. Additional features included autoregressive coefficients, variance of central frequency, peak-to-average power ratios, and complexity metrics such as fuzzy entropy, Rényi entropy, and entropy from singular value decomposition. Other measures captured signal stability and band-specific energy distributions. In total, fifty features were taken into account.

All features were computed for each HD-sEMG channel individually and then averaged across the 32 channels, providing a robust summary of the muscle activation pattern for each segment. To account for the different scales and units of the extracted features, which included statistical, frequency-based, and entropy-related measures, a z-score normalization was applied. This normalization was crucial to ensure that all features contributed equally to the subsequent analysis, avoiding dominance by those with larger numerical ranges.

A Principal Component Analysis (PCA) was then carried out on the normalized dataset to reduce dimensionality and enable a two-dimensional visualization of the feature space. The features contributing most to the first principal component (PC1) were waveform length, spectral entropy, frequency ratio, mean power ratio, and variance. The second principal component (PC2) was primarily influenced by the mean absolute value slope, mean power, mean frequency, mean of the absolute signal raised to the power 0.5, and Hjorth mobility. To further refine the analysis, we applied a feature selection method based on minimum redundancy–maximum relevance (mRMR), aiming to retain the most informative and least redundant features. This approach identified the set of fifteen features listed in Table 3.

### 2.7. Classification

Following the feature extraction and dimensionality reduction via PCA described in the previous subsection, a supervised classification was performed to assess the discriminative power of the selected feature subset in distinguishing PD patients from healthy control subjects. The classification was carried out using a Support Vector Machine (SVM) with an RBF kernel, applied to the combination of the three swallowing tasks (WT, GT, ST) and limited to the first and third principal components (PC1 and PC3).

Given the imbalance between the fifteen PD patients and seven control subjects, a balanced cross-validation strategy was adopted. Subjects were randomly partitioned into folds, each containing exactly two PD patients and two controls, ensuring class balance during training and testing. This process was repeated across 500 different random fold assignments to identify the most stable and effective partition.

We deliberately adopted classical and transparent machine learning techniques, including feature-based analysis, PCA, and SVM classification, rather than deep learning models which are black-boxes. So, each of the extracted features has a direct physiological interpretation related to muscle activation intensity, coordination, and spectral content, which can be intuitively understood by clinicians.

## 3. Results

As mentioned, a target of this work was to extract temporal and spectral features from the sEMG signal and identify possible direct correlations with specific PD scores and other aspecific scores. We focused on the following: (1) the MDS-UPDRS part III (evaluating the motion and overall condition); (2) the UPDRS axial score, calculated as the sum of the axial subitems of the MDS-UPDRS part III (more in detail, subitems 3.1, 3.2, 3.3, 3.9, 3.10, 3.11. 3.12, 3.13); (3) the SDQ total score, in addition to clinical-demographical information; (4) the current age; and (5) the disease duration.

### 3.1. Time Analysis

**Figure 3:**
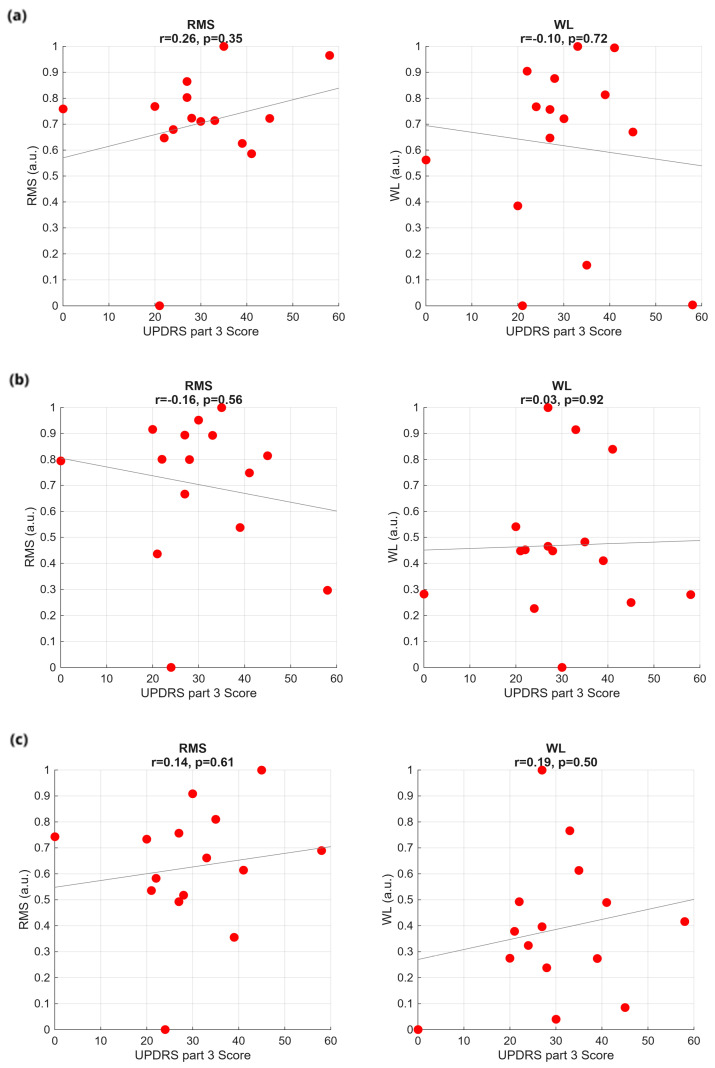
Time-domain features plotted against the UPDRS part III score for the three swallowing tasks: (**a**) WT, (**b**) GT, and (**c**) ST.

**Figure 4:**
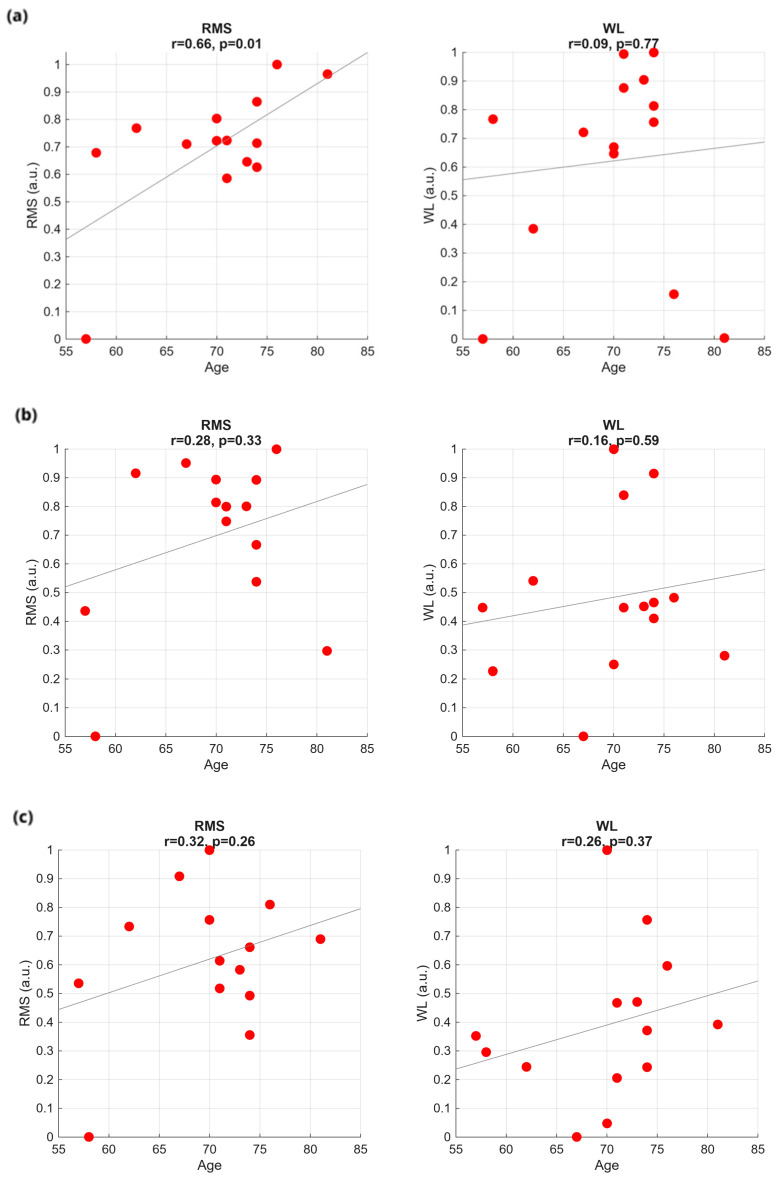
Time-domain features plotted against patient age for the three swallowing tasks: (**a**) WT, (**b**) GT, and (**c**) ST.

We performed a systematic investigation to find out correlations between the considered time features (RMS, WL, STD, AS) and the clinical and demographic data (the UPDRS part III, the UPDRS axial score, the SDQ, the subject age, the disease duration) for the three bolus consistencies. As a result, we can affirm that, in general, clinical and demographic data cannot be considered as clear and meaningful factors influencing the swallowing time features. A significant correlation was observed only between RMS in the WT and subject age (*p* = 0.01, r = 0.66). For clarity, we report in the following just a few results, without altering the general conclusion of the study. In particular, we show normalized RMS and WL for the three bolus consistencies, as a function of a specific score mostly representative of the PD, the UPDRS part III (Figure 3), and as a function of the aspecific score more frequently associated with dysphagia, the subject age (Figure 4).

All the sEMG features were calculated individually for each channel and then averaged over the 32 channels. In addition, the values were averaged over the three trials. So, for each patient, one value was obtained for each feature in the WT plot, in the GT plot, and in the ST plot. On top of the plots, we report the Pearson correlation coefficient value r and the associated *p*-value from Pearson’s correlation test. As one can see, data are widely scattered. The regression lines depicted inside the plots are mostly determined by singularities, rather than by true data trends.

### 3.2. Frequency Analysis

**Figure 5:**
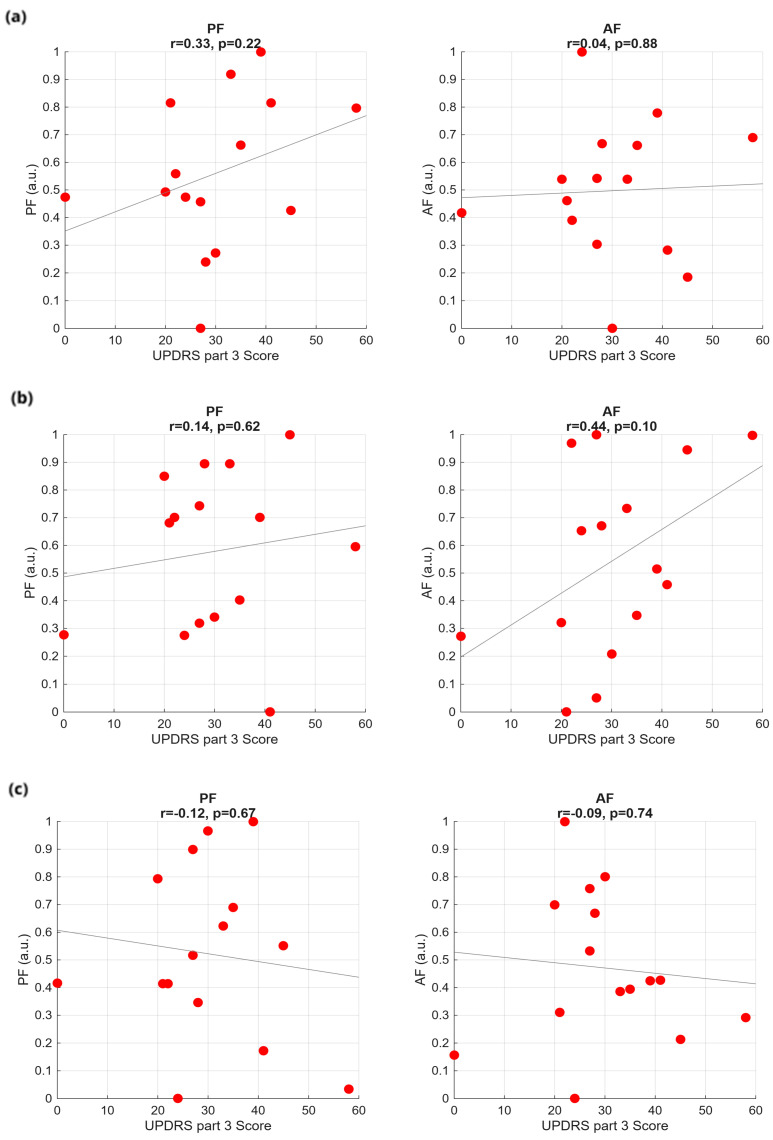
Frequency-domain features against UPDRS part III score for the three swallowing tasks: (**a**) WT test, (**b**) GT test, and (**c**) ST test.

**Figure 6:**
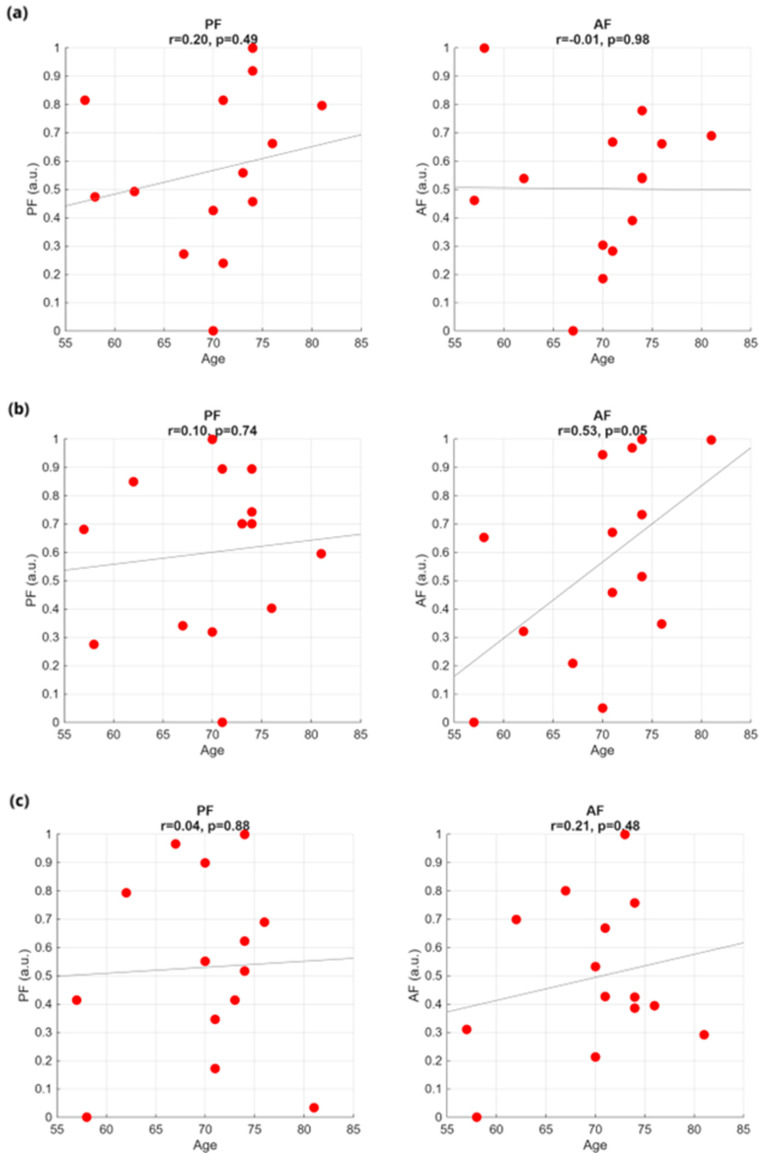
Frequency-domain features against patient age for the three swallowing tasks: (**a**) WT test, (**b**) GT test, and (**c**) ST test.

As in the time domain, no significant correlation is observed between the considered sEMG frequency features (PF, AF, TP) and the clinical and demographic data, independently of the bolus consistency. As an example, we show AF and TP as function of the UPDRS part III (Figure 5) and the subject age (Figure 6).

### 3.3. Principal Component Analysis

**Figure 7:**
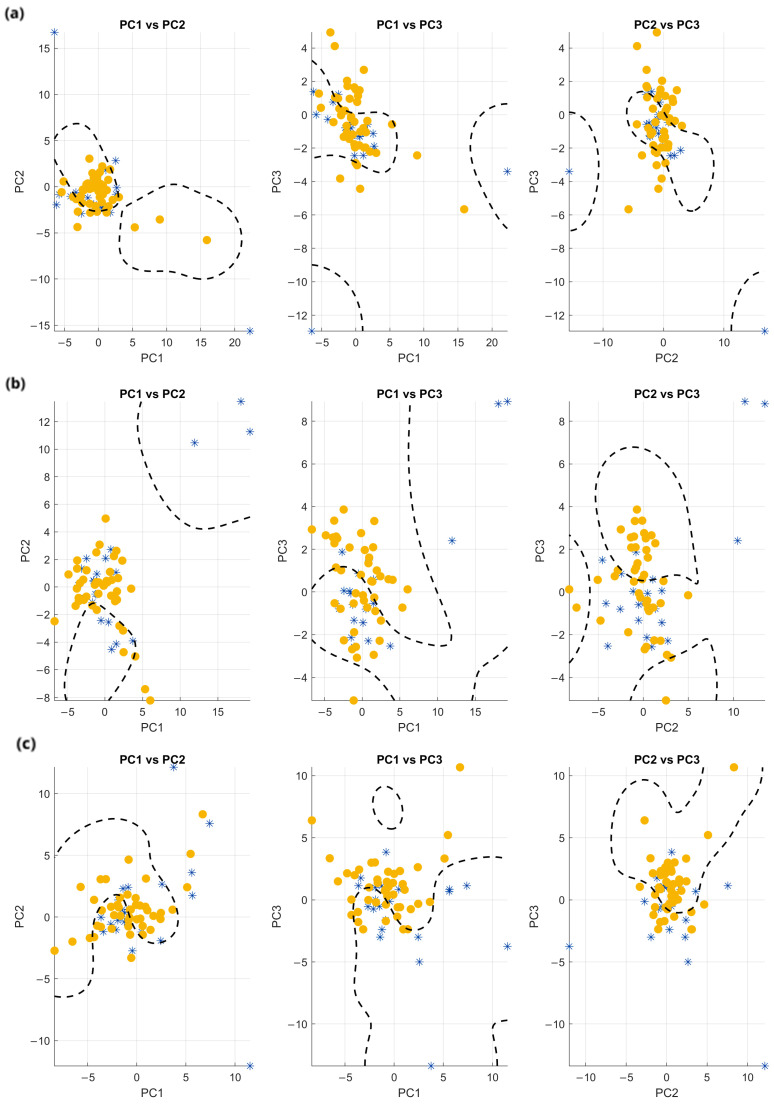
PCA projections onto the PC1–PC2, PC1–PC3, and PC2–PC3 planes for each task: (**a**) WT, (**b**) GT, and (**c**) ST. Yellow symbols represent patients, blue symbols represent controls. A dashed line in each subplot represents a non-linear SVM classifier with an RBF kernel trained on the corresponding PCA components.

PCA was first applied to the complete set of fifty features to explore whether the feature space allowed a natural separation between patients and controls. As shown in Figure 7, neither the 3D PCA plot nor its two-dimensional projections (PC1 vs. PC2, PC1 vs. PC3, PC2 vs. PC3) revealed a clear distinction between the two groups. Blue symbols represent controls and yellow symbols represent patients. In each subplot, a dashed line represents a non-linear SVM classifier with a radial basis function (RBF) kernel trained on the corresponding PCA components. Although this classifier is clearly overfitted to the data and not used for actual classification purposes, it is shown solely as a visual aid to highlight the potential boundaries between the two groups.

As one can see, from those plots it seems impossible to infer the existence of two separate clusters, probably because the set of features was redundant.

**Figure 8:**
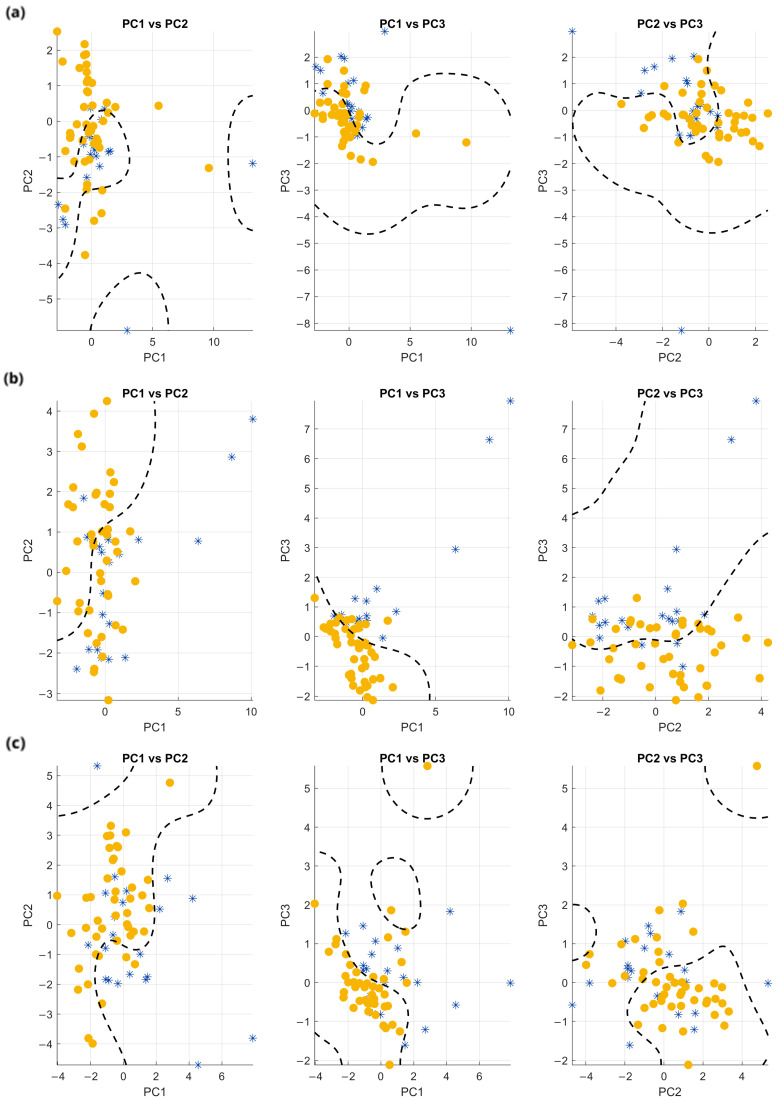
PCA projections onto the PC1–PC2, PC1–PC3, and PC2–PC3 planes for each task using the 15 most relevant features selected via mRMR: (**a**) WT, (**b**) GT, and (**c**) ST. Yellow symbols represent patients, blue symbols represent controls. A dashed line in each subplot represents a non-linear SVM classifier with an RBF kernel trained on the corresponding PCA components.

To address this, PCA was repeated using only the subset of fifteen features selected by the mRMR algorithm. When the PCA was repeated using only those selected features, the visualization showed a markedly improved separation between a group mainly composed of controls (blue symbols) and another group consisting mainly of patients (yellow symbols), demonstrating that a subset of carefully chosen features can carry stronger discriminatory power than the full feature set. Figure 8 reports PC1 vs. PC2, PC1 vs. PC3, and PC2 vs. PC3 plots relative to bolus with progressively increasing viscosity, from WT to ST.

Again, a dashed line in each subplot represents a non-linear SVM classifier with an RBF kernel trained on the corresponding PCA components. All data were used for training, with binary labels assigned to indicate patient or control status, corresponding to different symbol colors in the plot. The resulting decision boundaries were plotted with dashed lines on the three PCA projections of Figure 8. To account for the unbalanced number of subjects (15 patients vs. 7 controls), inverse class frequencies were used to weigh the training samples.

Noticeably, in all the subplots of Figure 8 two almost homogeneous clusters can be easily identified: one predominantly composed of controls (blue symbols) and the other of patients (yellow symbols), with the SVM line tracing clear separation boundaries. This visual impression is supported by a quantitative assessment of the SVM classification performance on the same data (i.e., overfitted by design, for visualization purposes only). For each projection and task, we computed standard classification metrics (accuracy, precision, recall, and F1-score), which are summarized in Table 4.

Interestingly, the best separation between patients and controls was clearly achieved in the PC1 vs. PC3 projection for all tasks (WT, GT, ST). In this plane, the SVM yielded the highest accuracy and F1-scores, indicating that it provides the clearest margin between the two groups. In general, we found accuracy to be around 80%, precision around 90%, and F1-score higher than 80%, independently of the bolus viscosity (as evidenced with bold character in Table 4).

The separation appears visually generally more distinct in the case of the solid swallowing test (ST), compared to WT and GT. This observation is confirmed by higher SVM metrics in Table 4. This can be due to the fact that ST is the more complex task of the three, with intense and prolonged muscular activation, which can penalize more severely patients with prodromal signs of swallowing impairment with respect to healthy subjects.

### 3.4. Classification

Supervised classification was performed using the SVM model trained on the selected mRMR features projected onto PC1 and PC3, combining data from the three swallowing tasks (WT, GT, ST).

The best-performing configuration, identified with seed 151, is detailed in Table 5 and showed excellent classification results across all evaluation metrics. This specific fold arrangement yielded a particularly strong balance between sensitivity and specificity, with a very high recall and overall accuracy. These results demonstrate that the proposed framework can reliably separate PD patients from healthy controls from the point of view of their swallowing-related sEMG features.

Table 5 also reports the average classification performance over all 500 seeds. While the mean results are significantly lower than the best case, they remain good to excellent across most metrics. This reduction in performance is not unexpected, as the clinical dataset does not provide a formal diagnosis of dysphagia. Some PD patients included in the study may currently exhibit normal or near-normal swallowing function and can therefore be misclassified as controls during testing. This intrinsic variability, due to the heterogeneity of swallowing impairment in PD, affects the overall classification reliability across random folds. Nonetheless, this variability opens up a clinically significant opportunity. Indeed, the fact that the classifier detects differences in muscle activation patterns among PD patients without clinically diagnosed dysphagia suggests that it may capture early neuromuscular alterations associated with swallowing dysfunction. Therefore, this HD-sEMG-based classification approach may serve as a valuable early screening tool to identify PD patients at increased risk of developing dysphagia before the onset of clinically detectable symptoms. By revealing latent or subclinical abnormalities in swallowing behavior, the method could support preventive clinical decision-making, including closer follow-up, early intervention, and longitudinal monitoring of progression.

## 4. Discussion

Dysphagia represents one of the most significant complications of PD, with important clinical and prognostic implications. Early identification of this symptom is essential to reduce the risk of respiratory and nutritional complications, improve patients’ quality of life, and optimize therapeutic management. In this context, the present study evaluated the effectiveness of a non-invasive wearable sensor system, combining accelerometers and surface electromyography, to objectively characterize swallowing in PD patients without clinically manifested dysphagia. Signals were recorded from a cohort of patients and healthy control subjects performing all the same swallowing test protocols, which included three tasks with different viscosity boluses. A number of relevant temporal and spectral features were extracted from the sEMG data. Subsequent filtering and processing by machine learning and Principal Component Analysis identified two distinct clusters, demonstrating the system capacity to discriminate functional swallowing profiles: one cluster was predominantly composed of controls and just a few patients, while most of the patients crowded the other cluster. The Support Vector Machine line traced a clear separation boundary between the two groups. The separation was more distinct in the case of the Solid bolus Task, probably due to the fact that the solid task is the more complex of the three, with intense and prolonged muscular activation, which can penalize more severely patients with prodromal signs of swallowing impairment with respect to healthy subjects. In general, we found excellent classification performance: accuracy and precision around 80%, and F1-score higher than 80%, for all the bolus viscosities.

Importantly, the proposed analysis pipeline was designed to remain interpretable and compatible with clinical reasoning. All extracted features have direct physiological meaning, reflecting muscle activation intensity, coordination, or frequency content and can therefore be easily understood by clinicians. The visual representation obtained through PCA further facilitates communication and interpretation of results in multidisciplinary contexts, supporting potential adoption in laboratory and pilot clinical settings.

The classifier revealed consistent differences in muscle activation patterns among PD patients, even in the absence of clinically diagnosed dysphagia. These findings suggest the presence of early neuromuscular alterations that cannot be captured through subjective reporting or standard clinical examination. Overall, the results support the feasibility of wearable sensor-based assessment as a reliable and non-invasive tool for detecting subclinical swallowing dysfunction in PD. Nevertheless, future studies involving multiple operators and diverse patient cohorts are warranted to rigorously evaluate the repeatability of these findings and to assess the wearability and user acceptability of the implemented acquisition devices.

In conclusion, the proposed approach is characterized by its non-invasiveness, ease of use, and potential adaptability for home monitoring. These features make its application particularly appealing not only in specialized clinical settings, but also for the development of telemedicine strategies and secondary prevention. Looking ahead, the integration of wearable technologies into the management of PD patients could provide valuable support for diagnostic and rehabilitative processes, contributing to a timelier, more personalized and continuous approach to dysphagia care and follow-up.

### Limitations of the Current Methodology and Perspectives

This study has some limitations. First, the patient sample size was relatively small. However, it should be noted that we exclusively enrolled individuals affected by PD, which substantially reduces the pool of eligible participants compared to the broader population of patients possibly suffering swallowing impairment [31,32]. Regarding the predominance of male participants, it should be noticed that it reflects the epidemiological characteristics of PD itself, showing a male-to-female incidence ratio ranging from approximately 1.3 to 2.0 [33]. One could argue that the sample size is small for a reliable machine learning analysis, and that the predominance of male participants prevents any gender-related inference. However, the good classification performance obtained with even such a small group supports the validity of the proposed methodology. Further studies involving larger and more balanced cohorts will hopefully strengthen these preliminary findings.

Moreover, this study represents a cross-sectional evaluation. Since dysphagia develops progressively and with substantial interindividual variability, longitudinal evidence is needed to confirm whether the identified electrophysiological patterns can reliably anticipate future swallowing impairment. The present work should therefore be considered a first step towards such longitudinal validation, which is already planned.

Finally, the 4 × 8 HD-sEMG grid was employed to capture the highest possible spatial resolution and to allow for the assessment of potential lateralization and axial symptomatology, topics that will be further explored for clinical purposes. Unfortunately, the grid is cumbersome and not ideal for daily or at-home use. However, the encouraging results obtained so far averaging features across the 32 channels may suggest that a reduced number of electrodes could achieve similar results, while being more practical. Future work will also integrate complementary modalities, such as speech analysis, which has recently emerged as a promising tool for the early detection and monitoring of dysphagia [34,35].

## Figures and Tables

**Table 1:** Information on the patients studied: gender, age, disease duration, clinical scores, therapy. Movement Disorder Society-Unified Parkinson’s Disease Rating Scale (MDS-UPDRS), Swallowing Disturbance Questionnaire (SDQ), Levodopa Equivalent Daily Dose (LEDD).

Patient ID	Gender	Age (Years)	Disease Duration (Years)	MDS-UPDRS Part III	MDS-UPDRS Axial Score	SDQ	LEDD (mg)
PT01	M	73	6	22	8	5	975
PT02	M	76	11	35	11	7	712
PT03	F	62	4	20	9	6	750
PT04	M	77	10	54	13	10	350
PT05	M	71	21	28	9	3	1219
PT06	M	58	5	24	7	3	600
PT07	F	74	14	39	16	6	1225
PT08	F	67	14	30	12	2	1092
PR02	F	74	3	27	4	13	300
PR03	M	81	23	58	20	15	655
PR04	M	70	15	45	16	9	1468
PR05	M	57	7	21	5	3	832
PR06	M	71	8	41	11	3	900
PR07	M	74	16	33	11	9	1200
PR08	M	58	13	28	16	4	1610

**Table 2:** Information on the studied healthy controls (HC): gender, age, Swallowing Disturbance Questionnaire (SDQ).

Patient ID	Gender	Age (Years)	SDQ
HC01	F	65	1
HC02	M	65	2
HC03	F	62	6
HC04	M	65	4
HC05	M	74	1
HC06	F	68	2
HC07	F	60	3

**Table 3:** Features selected through mRMR to reduce redundancy and enhance group separation.

N°	Features
1	Slope Sign Changes
2	Hjorth Complexity
3	Variance
4	Multiplied Power and Peak Amplitude
5	Singular Value Decomposition Entropy
6	Zero Crossings
7	Ratio of Mean Frequency to Median Frequency
8	Variance of Central Frequency
9	Median Frequency
10	Spectral Concentration Measure
11	Standard Deviation
12	Difference in Moments (4th–2nd)
13	Instantaneous Median Frequency
14	Mean Power Ratio
15	Mean Absolute Value

**Table 4:** SVM classification metrics computed in overfitting on the training data used for visual separation in PCA plots. The best-performing projection for each task is highlighted in bold.

Task	PCA Plane	Accuracy (%)	Precision (%)	Recall (%)	F1-Score (%)
WT	1–2	74.24	93.75	66.67	77.92
**WT**	**1–3**	**77.27**	**89.47**	**75.56**	**81.93**
WT	2–3	72.73	96.55	62.22	75.68
GT	1–2	61.54	91.30	47.73	62.69
**GT**	**1–3**	**81.54**	**90.00**	**81.82**	**85.71**
GT	2–3	73.85	90.91	68.18	77.92
ST	1–2	83.33	85.42	91.11	88.17
**ST**	**1–3**	**84.85**	**90.70**	**86.67**	**88.64**
ST	2–3	68.18	81.58	68.89	74.70

**Table 5:** Classification metrics averaged across 500 random fold initializations. Values are reported as mean ± standard deviation.

Metrics	Best Value (%) Seed (151)	Average on 500 Trials (%)	STD (%)
Accuracy	83.3	67.56	5.47
Precision	79.0	66.22	5.36
Recall	90.7	71.77	8.19
F1-score	84.5	68.68	5.62
Cohen’s kappa	0.67	0.35	0.11

## Data Availability

The dataset is not publicly available because it is currently being used by multiple research groups funded under the same project. Data sharing will be considered once all ongoing studies within the project have been completed.

## Abbreviations

The following abbreviations are used in this manuscript:

IEIIT	Institute of Electronics, Computer and Telecommunication Engineering
CNR	Consiglio Nazionale delle Ricerche
HD-sEMG	High-Density surface Electromyography
PD	Parkinson’s Disease
VFSS	Videofluoroscopic swallow study
FEES	Fiberoptic endoscopy evaluation of swallowing
IMU	Inertial measurement units
LDA	Linear Discriminant Analysis
HC	Healthy Controls
MDS	Movement Disorder Society
MoCA	Montreal Cognitive Assessment
MDS-UPDRS	Movement Disorder Society-Unified Parkinson’s Disease Rating Scale
FAB	Frontal Assessment Battery
SDQ	Swallowing Disturbance Questionnaire
CET	Comitato Etico Territoriale
LEDD	Levodopa Equivalent Daily Dose
WT	Water Task
GT	Gelled water Task
ST	Solid bolus Task
IQR	Interquartile range
MS	Mastication start
ME	Mastication end
SS	Swallowing start
SE	Swallowing end
RMS	Root mean square
WL	Waveform length
AS	Asymmetry index
DOS	Degree of Symmetry
FFT	Fast Fourier Transform
PF	Peak frequency
AF	Average frequency
TP	Total power
PCA	Principal Component Analysis
PC1	First principal component
PC2	Second principal component
PC3	Third principal component
mRMR	Minimum redundancy–maximum relevance
SVM	Support Vector Machine
RBF	Radial basis function
